# Magnitude and Predictors of Pneumonia among Under-Five Children in Ethiopia: A Systematic Review and Meta-Analysis

**DOI:** 10.1155/2020/1606783

**Published:** 2020-05-30

**Authors:** Yoseph Merkeb Alamneh, Fentahun Adane

**Affiliations:** Department of Biomedical Sciences, School of Medicine, Debre Markos University, Debre Markos, Ethiopia

## Abstract

**Background:**

Pneumonia is currently the leading cause of morbidity and mortality among under-five children in developing countries, including Ethiopia. Although these problems are easily preventable and treatable, it contributes to more than 18% of deaths of under-five children every year in Ethiopia. Regardless of these facts, there is a paucity of information regarding the magnitude and its predictors of pneumonia in Ethiopia. Therefore, the main objective of this review is to determine the pooled magnitude of pneumonia and its predictors among under-five children in Ethiopia.

**Methods:**

The international databases such as MEDLINE/PubMed, EMBASE, Google Scholar, and Science Direct were scientifically explored. Articles were also searched by examining the gray literature on institutional databases and by reviewing reference lists of already identified articles. We considered all primary studies reporting the magnitude of pneumonia among under-five children and its predictors in Ethiopia. We retrieved all necessary data by using a standardized data extraction format spreadsheet. STATA 14 statistical software was used to analyze the data, and Cochrane's Q test statistics and *I*^2^ test were used to assess the heterogeneity between the studies. Significant variability was found between the studies in such a way that a random-effect model was used.

**Result:**

The pooled magnitude of pneumonia among under-five children was 20.68% (*I*^2^ = 97.9%; *P* ≤ 0.001) out of 12 studies in Ethiopia. Children who have unvaccinated (OR = 2.45), food cooking in the main house (OR = 2.46), vitamin A supplementation status (OR = 2.85), malnutrition (OR = 2.98), mixed breastfeeding (OR = 2.46), and child history of respiratory tract infection (OR = 4.11) were potential determinates of pneumonia. *Conclusion and Recommendations*. This review showed that the magnitude of pneumonia was relatively high. Hence, appropriate intervention on potential determinates such as health education on exclusive breastfeeding and nutrition, place of food cooking, increased immunization and vitamin A supplementation, and early control of respiratory tract infection was recommended to prevent those risk factors.

## 1. Background

Pneumonia is defined as an acute lung parenchyma infection that can be caused by many pathogens such as viruses, bacteria, and fungi [1, 2]. Pneumonia is the leading cause of morbidity and mortality among under-five children [3]. In 2016, pneumonia accounted for approximately 16% of the 5.6 million under-five deaths, killing around 900,000 children worldwide [4]. In the same year, according to the WHO report, pneumonia accounted for approximately 16% of the estimated 5.9 million deaths among children under 5 years of age [5, 6]. Worldwide, the incidence of pneumonia in the under-five age group is estimated to be 0.29 and 0.05 episodes per child-year in developing and developed countries, respectively, of which about 7–13% are life-threatening and require hospitalization [7]. Sub-Saharan Africa bears the highest (50%) burden of the global under-five mortality rate of pneumonia [8]. Approximately 490,000 children under the age of five died of pneumonia in sub-Saharan Africa in 2016 [9]. Ethiopia is one of the SSA countries with the highest rates of pneumonia, with an estimated 3,370,000 children encounter pneumonia annually, which contributes to 18% of all causes of deaths of more than 40,000 under-five children every year [5].

Even though these deaths are easily treatable and preventable by cost-effective interventions such as immunization, proper nutrition, exclusive breastfeeding, zinc and vitamin A supplementation, appropriate complementary feeding, safe drinking water, and good sanitation and control of environmental factors, delays in recognizing pneumonia contribute to the highest to the death of under-five children in every year [10, 11]. Although the Ethiopian government has adopted and implemented different strategies to reduce pneumonia morbidity and mortalities, the burden and severity of childhood pneumonia are high and continue to cause common mortality of children due to limited coverage and affordability of effective preventive interventions like immunization, and lack of good access to care and unavailability of effective management strategies which calls for innovative strategies that will come about only through systematic research studies [12, 13]. Although the magnitude of pneumonia among under-five children in Ethiopia has improved due to different interventions, it remains the major cause of death among under-five children.

In Ethiopia, contradicted and inconsistent studies have been conducted to assess the magnitude and predictors of pneumonia among under-five children. The impact is still higher and with abundant discrepancy and inconstancy across regions related to assessing the magnitude and predictors of pneumonia. Data on the magnitude of pneumonia and its related risk factors are important for planning child healthcare services but scarce in Ethiopia. Regardless of these facts, in Ethiopia, there is a paucity of information regarding the magnitude and predictors of pneumonia. Thus, assessing the pooled magnitude of pneumonia and its predictors at a country level is essential and will provide an overall figure with better estimation. Therefore, the main objective of this systematic review and meta-analysis was to estimate the pooled magnitude of pneumonia and its predictors in Ethiopia. Findings obtained from this review will contribute to evidence for policymakers and program planners who are working at various levels on the area to inform, plan, implement, and evaluate health promotion policies and strategies. The study also will provide baseline information for researchers.

## 2. Materials and Methods

### 2.1. Searching Strategies and Study Design

This review was considered to evaluate the combined magnitude and predictors of pneumonia among under-five children in Ethiopia. In this review, we searched databases without limit to the date of publication and study design. To confirm the scientific accuracy, the Preferred Reporting Items of Systematic Reviews and Meta-Analysis Protocol (PRISMA-P) guideline was used [14]. The international databases include MEDLINE/PubMed, EMBASE, Web of Sciences, Scopus, Google Scholar, Science Direct, and Cochrane Library were scientifically explored. Also, articles were searched by examining the gray literature on institutional databases and by reviewing reference lists of already identified articles to retrieve additional articles and included the articles relevant to our topic of review. Unpublished studies were retrieved from the official websites of international and local organizations and universities.

The search was performed by keywords and Medical Subject Headings (MeSH) terms. We used the search terms independently and/or in combination using “OR,” “AND,” or “NOT.” The key terms used for the database searches were “magnitude” OR “Epidemiology” AND “Children” AND/OR “Under Five Children” AND/OR “Childhood” AND/OR “factors” AND/OR “associated factors” AND/OR “risk factors” AND/OR “determinants” AND/OR “predictors” AND” Ethiopia.” These search terms have been predefined in order to allow a comprehensive search strategy that includes all fields in records and Medical Subject Headings (MeSH terms) to extend the search in advanced PubMed search (Table 1). All articles conducted from September 30, 2019, up to November 30, 2019, and all accessible studies up to November 30, 2019, were incorporated in our meta-analysis and systematic review.

### 2.2. Study Identification, Selection, and Eligibility Criteria

This review included studies that were reported the magnitude of pneumonia and predictors among under-five children in Ethiopia. All full-text, English-language articles for studies conducted in Ethiopia, published between 2014 and 2019, in peer-reviewed journals or published in gray literature were eligible for inclusion. All studies which reported pneumonia magnitude and predictors among under-five children in Ethiopia were eligible for inclusion. Studies which did not report quantitatively specific pneumonia outcomes were excluded. We also excluded the primary studies, inaccessible of the full-text article after contacting the primary author two times through email.

### 2.3. Outcome of Interest

The main objective of this study was to determine the pooled prevalence of pneumonia among under-five children in Ethiopia. The prevalence was calculated as the number in the study of under-five children with pneumonia divided by the total number in a study of under-five children multiplied by 100. We collected data on factors considered to be related to pneumonia in the literature for the study of the secondary outcomes (factors associated with pneumonia), such as vaccination, place of food cooking, Vit A supplementations, nutritional status, exclusive breastfeeding, and respiratory tract infection. Data from the primary studies were collected in the form of two-by-two tables in the analysis of pneumonia-related factors, and odds ratio (OR) was calculated to determine the relationship between each of the independent variables and pneumonia.

### 2.4. Data Extraction and Synthesis

Data were retrieved by two independent reviewers (YMA and FA) by using a standardized data extraction spreadsheet format. The data abstraction format includes the author, the study year, region of study setting (region and rural or urban), study design, sample size, magnitude, and predictor variables. Any disagreements during the extraction process were solved by consensus between the reviewers. If we got incomplete data, we excluded the study after two attempts were made to contact the corresponding author by email. Also, the two authors (YMA and FA) performed the quality assessment of studies independently. Any discrepancy was resolved by discussion and agreement.

### 2.5. Quality Assessment (Appraisal) and Risk of Bias Assessment

Duplicate articles were removed manually using Endnote (version X8) after combining the database search results. To assess the quality of each study, Joanna Briggs Institute Meta-Analysis of Statistics Assessment and Review Instrument (JBI-MAStARI) adapted for both cross-sectional and case-control study design was used [15]. The original study was assessed by two reviewers independently and any disagreement between the reviewers was solved by taking the mean score of the two reviewers. Finally, the original studies which score five and above were included in the final review.

### 2.6. Statistical Methods and Analysis

For further analysis, we imported the data into the STATA version 14.0 statistical software after extracting the data using Microsoft Excel format.

Using the binomial distribution formula, the standard error was calculated for each study. The overall pooled magnitude of pneumonia among under-five children in Ethiopia was calculated using the standard error (SE) from each study. The pooled magnitude of pneumonia with 95% CI was presented using forest plots and odds ratio (OR) with 95% CI was also presented in forest plot to show the predictors with pneumonia.

### 2.7. Heterogeneity

We identify the heterogeneity between the studies using Cochrane's Q statistics (chi-square), inverse variance (*I*^2^), and *P* values [16]. Heterogeneity or variation across studies was assessed using the inverse variance (*I*^2^) and Cochrane's Q statistic at 25%, 50%, and 75% was used to declare as low, moderate, and high heterogeneity, respectively [16]. Also, we used a forest plot to detect the presence of heterogeneity. Furthermore, subgroup analysis was conducted by region of the study, study period, and sample size, and meta-regression was used to identify the possible source of heterogeneity.

### 2.8. Publication Bias

The evidence of publication bias was checked using funnel plot symmetry. Besides, the statistical significance of publication bias was assessed using both Egger's and Begg's test; subsequently, a trim-and-fill analysis was performed, and the *P* value of less than 5% was used to declare the presence of publication bias [17, 18].

## 3. Results

### 3.1. Selection and Identification of Original Studies

**Figure 1:**
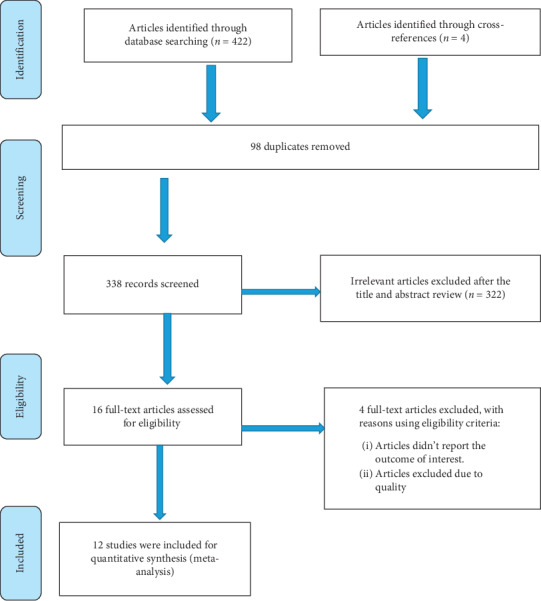
PRISMA flow diagram of included studies to estimate the magnitude of pneumonia and its predictors among under-five children in Ethiopia from 2014 up to 2019.

A total of 426 articles were generated in the search and desk review database. Of these, 422 articles were retrieved from PubMed, Google Scholar, and other electronic databases. The remaining 4 were identified in institutional repositories. After we checked the titles and abstracts, 98 studies were removed due to duplication. In screening, 312 studies were excluded from the remaining 328 studies after reviewing the title and the abstract because their findings were not reported. The abstracts and full texts of the remaining 16 articles were assessed and screened for eligibility criteria. Four additional articles were excluded because the outcome of the interest was not reported. Finally, 12 studies fulfilled the eligibility criteria and included in this systematic review and meta-analysis (Figure 1).

### 3.2. Characteristics of the Studies

In this review, a total of twelve articles met the inclusion criteria. A total of 4598 study participants included in this systematic review with an estimated sample size ranging from 206 to 558 in SNNP [19, 20] in individual studies, and all the included studies were published between 2015 and 2019. Both cross-sectional and case-control studies were included in the current review.

In this meta-analysis and systematic review, of the total 12 included studies, four studies were conducted from Amhara region [2, 21–23], four studies from Oromia region [24–27], two studies from SNNP [19, 20], and two studies from Addis Ababa [28, 29] (Table 2).

### 3.3. Meta-Analysis

#### 3.3.1. Magnitude of Pneumonia among Under-Five Children in Ethiopia

**Figure 2:**
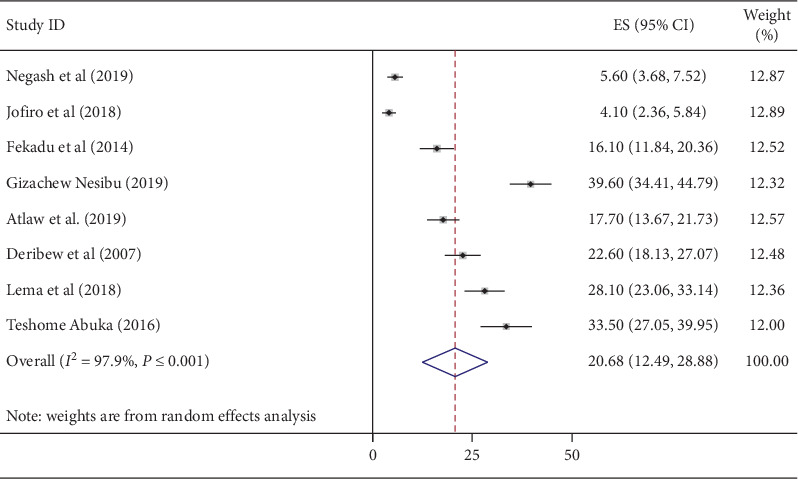
Forest plot showing the pooled magnitude of pneumonia among under-five children in Ethiopia from 2014 up to 2019.

The magnitude of pneumonia among under-five children in Ethiopia from included studies ranged between 4.1% and 39.6% [23, 29]. As indicated in the forest plot below, the pooled magnitude of pneumonia among under-five children from 12 studies in Ethiopia was 20.68% (95% CI: 12.49, 28.88). We identified a high and significant heterogeneity between studies (*I*^2^ = 97.9%; *P* ≤ 0.001), indicating great variability in the magnitude of pneumonia across studies; random-effect analysis model was used to estimate the pooled magnitude of pneumonia among under-five children in Ethiopia (Figure 2).

### 3.4. Publication Bias

Publication bias was observed using both Begg's and Egger's test [17, 18], and the value was found to be significant at a *P* value of 0.004 and 0.020, respectively. These tests showed that there was statistical evidence of publication bias at a *P* value of less than 0.05. By considering publication bias, trim-and-fill meta-analysis [31] was done to account for publication bias. However, based on this analysis, the prevalence of pneumonia among under-five children was 20.68%, and no significant change was observed as compared with the main meta-analysis.

### 3.5. Subgroup Analysis

Due to considerable heterogeneity in this review, subgroup analysis was done to identify the source of heterogeneity based on the region and sample size. Subgroup analysis by region reports the pooled prevalence of pneumonia among under-five children was higher in the Amhara region (27.80 %) followed by the Oromia region (22.65 %). Subgroup analysis was also carried out based on the sample size, the sample size was determined on the basis of the mean sample size of the included studies, and according to this analysis, the prevalence rates of 24.72% and 4.79% were revealed from sample size <384 and sample size ≥384, respectively (Table 3).

### 3.6. Sensitivity

A sensitivity analysis was done to identify outlier studies. According to the analysis, no influential studies were detected so that all of the studies were included in the final analysis.

### 3.7. Meta-Regression

Besides subgroup analysis and publication bias, univariate and categorical meta-regression analysis was performed undertaken by considering both continuous and categorical data for the included studies to identify sources of heterogeneity for the pooled prevalence. In meta-regression analysis, sample size, publication year, and study regions were considered for each study. However, there was no statistical significance value from the meta-regression analysis. Hence, the pooled prevalence of pneumonia among under-five children was not associated with sample size, publication year, and study region (Table 4).

### 3.8. Predictors of Pneumonia among Under-Five Children

In this review, a total of 12 studies were included for the analysis of predictors of pneumonia among under-five children. We identified six main associated factors with the pooled odds ratio ranging from 2.45 to 4.11. These predictors were vaccination, place of food cooking, vitamin A supplementations, nutritional status, exclusive breastfeeding, and respiratory tract infection. Unvaccinated children were 2.45 times more likely to have pneumonia as compared to children who fully vaccinated (OR = 2.45; 95% CI (1.13, 5.31)). This result showed that children from the household who used the main house for a place of cooking were 2.46 times more likely to develop pneumonia as compared to those from household cook food in the kitchen (OR = 2.46; 95% CI (1.66, 3.66)). Children who were not ever supplemented with vitamin A were 2.85 times more likely to develop pneumonia as compared to children who got vitamin A supplementation (OR = 2.85; 95% CI (1.36, 5.56)). This review revealed that children who had MUAC less than 11.5 cm were 2.98 times more likely to develop pneumonia than children who had MUAC greater than or equal to 11.5 (OR = 2.98; 95% CI (1.84, 4.84)). Besides, the odds of pneumonia among under-five children who had not got exclusive breastfeeding were 2.46 times higher risk to develop pneumonia as compared to children who had got exclusive breastfeeding during the first six 6 months (OR = 2.46; 95% CI (1.35, 4.47)). According to this review, children who had a history of respiratory tract infection were 4.11 times more likely to develop pneumonia compared to those who had no respiratory tract infection (OR = 4.11; 95% CI (1.98, 8.52)) (Table 5).

## 4. Discussion

Currently, pneumonia among under-five children is a driving cause of morbidity and mortality in middle- and low-income countries including Ethiopia. This research is essential to understand the prevalence of pneumonia among under-five children and the factors that affect them in order to intervene to increase patients, families, and communities' satisfaction with health services, and to set criteria for enhancing the quality of under-five healthcare facilities. Assessing the prevalence and its risk factors of pneumonia is essential for proper planning of child healthcare services, for proper management and prevention strategy. In this meta-analysis, we systematically reviewed studies assessing pneumonia and associated factors among under-five children in Ethiopia. As far as our knowledge, there is no previous review of the issue. Thus, this systematic review and meta-analysis aimed at estimating the pooled prevalence of pneumonia and its predictors in Ethiopia. The pooled prevalence of pneumonia among under-five children from 12 included studies in Ethiopia was 20.68% (95% CI: 12.49, 28.88) (*I*^2^ = 97.9%; *P* ≤ 0.001). This finding reported higher values than other studies done in Austria (4.1%) [30], in Mali (6.7%) [31], and in Kenya (6.9%) [32]. In contrast to this, our finding reported higher values than the studies done in Bangladesh (21.3%) [33], in Nigeria (31.6%) [34], and in Uganda (53.7%) [35]. This discrepancy may be due to socioeconomic and sociodemographic vitiations, the difference in the study setting, seasonal variation, inaccessibility and provision of vitamin A supplementation and immunization, lack of confirmatory laboratories, and imaging investigations. Thus, despite advances in preventive and prevention approaches, in low- and middle-income countries, pneumonia remains the leading cause of childhood mortality and hospitalization. The widespread acceptance of currently effective pneumonia prevention and management strategies remains a problem in many low- and middle-income countries [36]. This review showed that children from the household who used charcoal and main house for a place of cooking were more than two times more likely to develop pneumonia than those who did not use it. This finding was supported by other studies [21, 37, 38]. The possible explanations could be high indoor air pollution may adversely affect host defenses of the respiratory tract against pathogens. This finding also supported by the UNICEF report confirmed that exposing children to household air pollution like solid fuels during cooking food in the main house doubles their risk of pneumonia [39]. Children who were not ever supplemented with vitamin A were almost three times more likely to develop pneumonia as compared to children who got vitamin A supplementation. This finding is in agreement with that of a study done in Rwanda [40]. This may due to the fact that the role of vitamin A is essential in the growth and development of respiratory epithelial cells and lung tissue [41]. Our study finding indicated that unvaccinated children were 2.45 times more vulnerable to develop pneumonia as compared to children who fully vaccinated which were supported by the study undertaken in India [42]. This could be justified that vaccinations and immunizations prevent children from developing infections that can lead to pneumonia as a complication [43]. Also, the odds of pneumonia among under-five children who had not got exclusive breastfeeding were 2.46 times higher risk to develop pneumonia as compared to children who had got exclusive breastfeeding during the first six 6 months. This result is in agreement with the UNICEF report [39]. This review revealed that children who had MUAC less than 11.5 cm were three times more likely to develop pneumonia than children who had MUAC greater than or equal to 11.5. It was supported by a study conducted in India [44]. This might have explained that undernutrition which weakens the child's overall immune system and undernourished children have weakened respiratory muscles, which inhibits them from adequately clearing secretions found in their respiratory tract makes the child vulnerable to pneumonia [39]. According to this review, children who had a history of respiratory tract infection were 4.11 times more likely to develop pneumonia compared to those who had no respiratory tract infection which was supported by the studies done in Kenya and the Netherlands [45, 46]. Infections in the upper respiratory tract are very infectious and can quickly be spread to children from household contacts. These infections are mostly viral in origin and cause pneumonia in children [47].

### 4.1. Limitations

By including data from unpublished studies and gray literature, this meta-analysis and systematic review could account for publishing bias due to underreporting negative results, which contributes to bias in meta-analysis, thus misinforming researchers and policymakers. Furthermore, since this meta-analysis included accessible research recorded from a small number of regions in Ethiopia, the various areas in the nation may be underrepresented.

## 5. Conclusion

This review showed that the prevalence rate of pneumonia was relatively high in Ethiopia compared to other studies' reports from developing countries. Vitamin A supplementation, vaccination status, malnutrition, history of child respiratory tract infection, place of food cooking, and breastfeeding status during 6 months were independent potential predictors of under-five pneumonia. Hence, appropriate intervention on potential determinates such as health education on exclusive breastfeeding and nutrition, place of food cooking, increased immunization and vitamin A supplementation, and early control of respiratory tract infection was recommended to prevent those risk factors.

## Figures and Tables

**Table 1:** Example of MEDLINE/PubMed and Google Scholar database searches to analyze the magnitude and predictors of pneumonia among under-five children in Ethiopia.

Databases	Searching terms	Number of studies
Google Scholar	magnitude” “Epidemiology” “Children” “Under Five Children” “Childhood” “factors” AND/OR “associated factors” “risk factors” “determinants” “predictors” “Ethiopia”	125

MEDLINE/PubMed	((“pneumonia”[MeSH Terms] OR “pneumonia”[All Fields]) AND (“Ethiopia”[MeSH Terms] OR “Ethiopia”[All Fields])) AND (“child”[MeSH Terms] OR “child”[All Fields] OR “children”[All Fields])	47

From other databases		250

Total retrieved articles		422

Finally full articles relevant to our review		12

**Table 2:** Characteristics of 12 studies reporting the magnitude of pneumonia and its predictors among under-five children in Ethiopia from 2014 up to 2019.

SN	Author	Publication year	Region	Study area	Study design	Sample size	Types of study	Quality score
1	Negash et al	2019	Addis Ababa	Addis Ababa	Cross-sectional	549	Institutional-based	7
2	Jofiro et al	2018	Addis Ababa	Addis Ababa	Cross-sectional	499	Institutional-based	8
3	Fekadu et al	2014	Amhara	Este Town	Cross-sectional	286	Community-based	6
4	A. F. Dadi et al	2014	Amhara	Oromia Zone	Case-control	356	Institutional-based	7
5	GIZACHEW NESIBU	2019	Amhara	Debre Berhan	Cross-sectional	341	Institutional-based	6
6	Yordanos Markos et al	2019	Amhara	Gondar	Case-control	435	Institutional-based	7
7	Atlaw D, et al.	2019	Oromia	Arsi Zone	Cross-sectional	344	Community-based	8
8	A. Deribew et al	2007	Oromia	Gilgel Gibe	Case-control	336	Institutional-based	7
9	Lema et al	2018	Oromia	Jimma Zone	Cross-sectional	306	Institutional-based	8
10	Geleta et al	2016	Oromia	Kersa	Case-control	382	Institutional-based	7
11	Workineh et al	2017	SNNP	Gamo Gofa Zone	Case-control	558	Institutional-based	5
12	Teshome Abuka	2016	SNNP	Sidama Zone	Cross-sectional	206	Institutional-based	7

**Table 3:** Subgroup analysis which describes the pooled magnitude of pneumonia among under-five children in Ethiopia, 2019.

Subgroup		Included studies	Prevalence (95% CI)	Heterogeneity statistics	*P* value	*I* ^2^ (%)	Tau-squared
Sample size	<384	5	24.716 (16.829, 32.602)	59.84	0.000	93.3	75.4120
	≥384	2	4.792 (3.326, 6.257)	1.29	0.257	22.2	0.2496

Region	Addis Ababa	2	4.792 (3.326, 6.257)	1.29	0.257	22.2	0.2496
	Amhara	2	27.801(4.772, 50.831)	47.05	0.000	97.9	270.2563
	Oromia	3	22.646 (16.817, 28.475)	10.07	0.007	80.1	21.2225

**Table 4:** Univariate and categorical meta-regression for the included studies to identify the source of heterogeneity for the magnitude of pneumonia among under-five children in Ethiopia.

Variables	Characteristics	Coefficient	*P* value
Year	Publication	−0.2216302	0.865

Sample size		−0.0856182	0.027

Region	Amhara	Reference	Reference
	Addis Ababa	−22.83555	0.064
	Oromia	−4.9355	0.584
	SNNP	5.815305	0.636

**Table 5:** Identified predictors of pneumonia among under-five children in Ethiopia, 2019.

No.	Variables	OR (95% CI)
1.	Children who unvaccinated	2.45 (1.13, 5.31)
2.	Food cooking in the main house	2.46 (1.66, 3.66)
3.	Lack of vitamin A supplementation	2.85 (1.36, 5.56)
4.	Malnutrition	2.98 (1.84, 4.84)
5.	Mixed breast feeding	2.46 (1.35, 4.47)
6.	Child history of respiratory tract infection	4.11 (1.98, 8.52)

## Abbreviations

CI: Confidence interval
OR: Odds ratio
SSA: Sub-Saharan Africa
SNNP: South Nations Nationalities of People
U5M: Under-five mortalities
WHO: World Health Organization
MUAC: Middle upper arm circumference.
